# Supplementary material for: Signatures of Positive Selection in the Genome of Apis mellifera carnica: A Subspecies of European Honeybees
Source: Life (Basel). 2022 Oct 19;12(10):1642. doi: 10.3390/life12101642 (PMC9605442; doi:10.3390/life12101642)
Supplement: Supplementary file 1 [file life-12-01642-s001.zip › life-1919730-supplementary.pdf]

Signatures of positive selection in the genome of *Apis mellifera carnica*: a subspecies of European honey bees

Qiang Huang[1,2], Yong-Qiang Zhu[3], Bertrand Fouks[4], Xu-Jiang He[1,2], Qing-Sheng Niu[5], Hua-Jun Zheng[*3], Zhi-Jiang Zeng[*1,2]

[1] Honeybee Research Institute, Jiangxi Agricultural University, Zhimin Ave. 1101, Nanchang, 330045, China.

[2] Jiangxi Province Key laboratory of Honeybee Biology and Beekeeping, Jiangxi Agricultural University, Zhimin Ave. 1101, Nanchang, 330045, China.

[3] Shanghai-MOST Key Laboratory of Health and Disease Genomics, Chinese National Human Genome Center at Shanghai and Shanghai Institute for Biomedical and Pharmaceutical Technologies, Shanghai, China.

[4] Institute for Evolution and Biodiversity, Molecular Evolution and Bioinformatics, Westfälische Wilhelms Universität, Germany

[5] Apiculture Science Institute of Jinlin Province, Yuanlin Rd. 132108, Jinlin, China.

1. Results

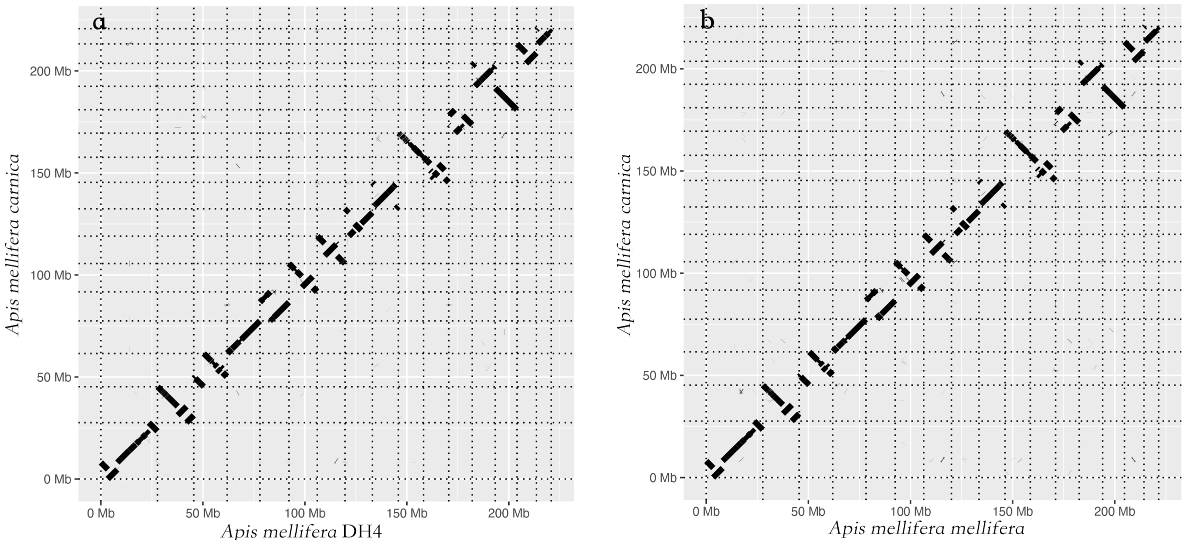

FigureS1 Dot plot of among three honey bee subspecies. The honey bee *Apis mellifera carnica* showed frame shifts and inversion compared with *A. mellifera* DH4 and *A. mellifera mellifera*.

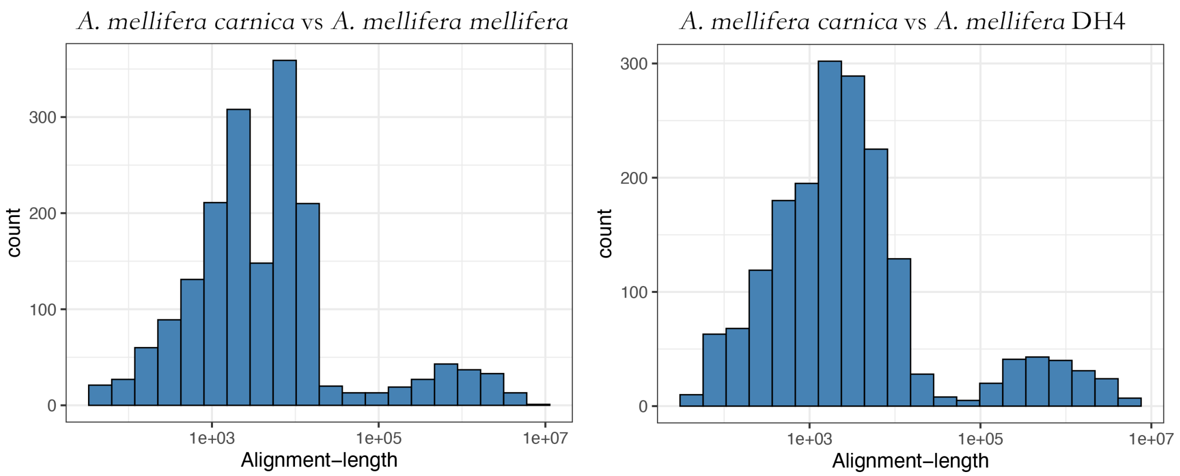

FigureS2 Aligned sequences between the genomes. The average length of the aligned region was 103 Kbp and 108 Kbp for *A. mellifera mellifera* and *A. mellifera* DH4 respectively

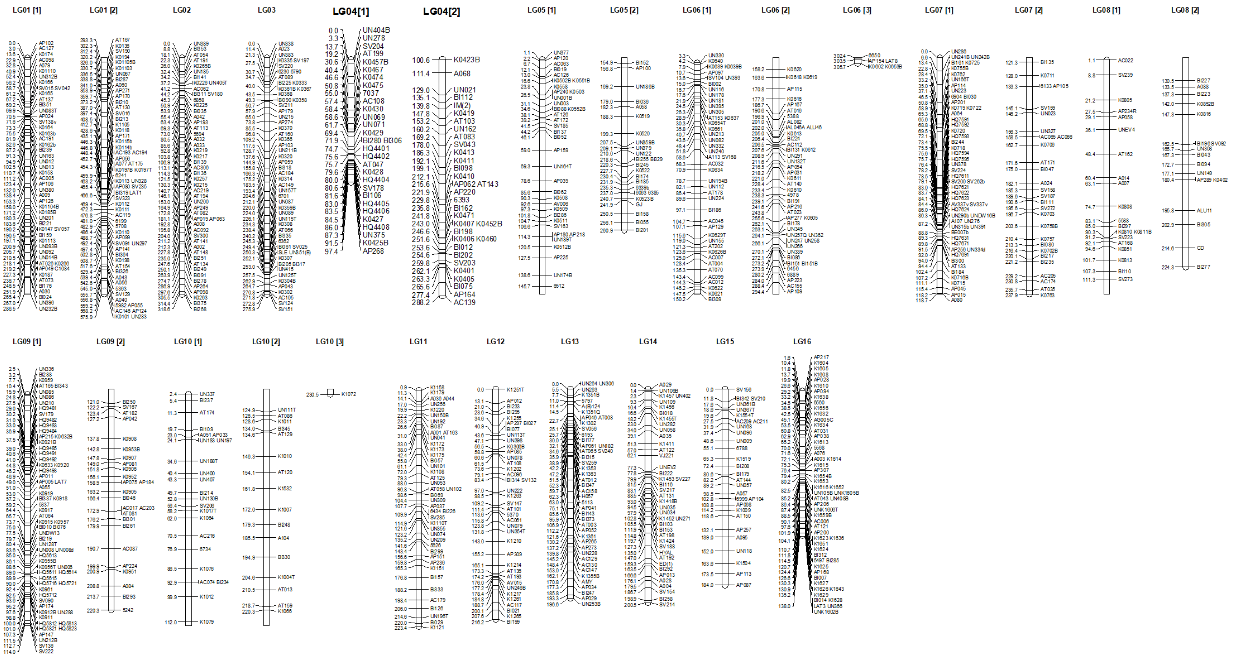

Figure S3 linkage map markers using to align among the three honey bee genomes.

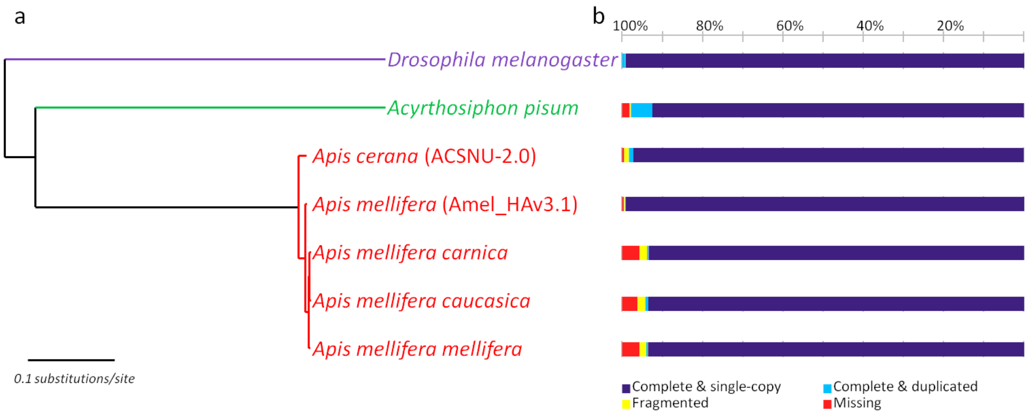

Figure S4 Phylogenetic tree and estimated completeness of the studies 7 genomes. (a) The phylogenetic tree was constructed on protein sequences of 874 single-copy orthologs shared among all 7 genomes. All nodes were 100% bootstrap supported. *D. melanogaster* was used to root the tree. (b) Completeness of predicted protein sets of each genome was assessed by aligning to the arthropod BUSCOs. For *A. mellifera carnica*, 93.53% of complete BUSCOs were found, which suggest the assembly is complete.

Table S1: Number of positively selected genes in each *A. mellifera* subspecies tested and as well as the *A. mellifera* branch and number of overlapping genes under positive selection in more than one branch.

	A. mellifera carnica	***A. mellifera ligustica***	***A. mellifera mellifera***	***A. mellifera caucasica***	***Apis mellifera spp.***
A. mellifera carnica	78	7	7	1	3
A. mellifera ligustica		114	1	0	11
A. mellifera mellifera			27	0	0
A. mellifera caucasica				10	0
Apis mellifera spp.					45
